# COVID-19: Pandemic burden in Sub-Saharan Africa and the right to health – The need for advocacy in the face of growing privatisation

**DOI:** 10.4102/phcfm.v12i1.2476

**Published:** 2020-09-14

**Authors:** Tshegofatso J. Sehoole

**Affiliations:** 1 Centre for Human Rights, Faculty of Law, University of Pretoria, Pretoria, South Africa

**Keywords:** privatisation, health, human rights, COVID-19, pandemic

## Abstract

For Africa, the backdrop^1^ against which COVID-19 emerged is a stark one. Although sub-Saharan Africa accounts for 11% of the world’s population, it bears 24% of the global disease burden. The continent is home to 60% of the people with human immunodeficiency virus (HIV), and over 90% of malarial patients. In this region, infectious diseases such as malaria and HIV cause 69% of deaths. As states respond to COVID-19, we need to keep our eyes open to what effective responses are notifying us about our healthcare systems, so that we can craft sustainable interventions as a result and uphold the right to health. This is especially true in the light of the ongoing nature of pandemics on the continent, making urgent the need to maximise the value of our health system and its resources, as we seek lasting transformation.

In 2018, the United Nations (UN) Special Rapporteur on extreme poverty criticised^2^ the extent to which the World Bank, the International Monetary Fund (IMF) and the UN have aggressively promoted privatisation of basic services, without regard to the human rights implications or consequences for the poor. The International Finance Corporation (IFC) found^3^ that the private sector already delivers about half of Africa’s health products and services, with private sector providers and for-profit providers (who garner 65% of healthcare expenditure) filling an important medical need for poor and rural populations that is not met by the public sector. The World Bank contends^4^ that by 2030, Africa might have 90% of the world’s poor. An analysis^5^ of the distributional impact of privatisation shows that it has disproportionately impacted the poor due to their higher financial vulnerability, and the lack of focus on bridging entrenched barriers to healthcare access.

Despite the impact of market conditions on human rights, governments are often encouraged^6^ to provide subsidies and tax breaks to attract private providers, which depletes revenues available for and is associated with a decline in investment in public health. Proponents of this approach assert the necessity of such investments to alleviating pressure from overwhelmed and ill-managed public sectors. This is despite the increase in private healthcare drawing needed healthcare workers away^6^ from the public system and multiplying catastrophic healthcare spending that exposes^7^ communities to severe financial risk. This further entrenches inequality, thereby weakening provision of universal healthcare. To be sure, although the private sector can and does play a valuable role in health, given the market failures in the health sector, it is unlikely that investments in private health (in and of themselves) generate better results than do public ones.

Privatised healthcare results in a highly regressive lack of cross-subsidisation from (usually low-risk) persons who are healthy and wealthy, to the (usually high-risk) sick and impoverished. For example, South Africa spends 42% of its total health expenditure on voluntary private health insurance – the highest percentage in the world^5^, for a scheme that covers only 16% of the population, leading to unequal access to healthcare among different socio-economic and racial groups. This unjust situation has prompted varied state responses in dealing with COVID-19 in an environment where there is enhanced socio-political pressure and will to act. Spain^8^ – one of the worst affected countries outside China, second only to Italy in Europe – nationalised all its hospitals and healthcare providers. On the other hand, South Africa entered into negotiations^9^ with and requested a voluntary assistance from the private sector. Although each state has discretion in its economic and social policies and governance, there is no discretion on a state’s obligation to uphold human rights. An economic assessment of the outbreak of Ebola reveals that countries most affected had been urged in the past to prioritise conventional privatisation and deregulation of macro-economic liberalisation policies, but there was no similar support to build strong public health systems as a development imperative.^10^ COVID-19 further demonstrates the incapability of the market as the only force in fulfilling universal public health needs and also the state’s responsibility to mitigate the effects of privatised healthcare to give effect to the rights to life and health, especially for the most vulnerable, through mobilising the private health sector in service of public health goals.

In light of this, states are bound^11^ by international laws to ensure that privatisation does not constitute a threat to the availability, accessibility, acceptability and quality of healthcare facilities, goods and services. In addition, the African Commission on Human and People’s Rights has called^12^ on states to address with all appropriate measures their obligations in relation to the full realisation of the right to health, through tackling constraints and damaging acts that result from the privatisation, although the commission is still in the process of developing standards that would guide implementation and adherence. Unfortunately, African states have to address this in a context where global economic institutions working towards development (namely the IMF and World Bank) are mitigating the effects of an inherently highly unequal global economic system in ways that keep populations in developing countries permanently trapped^13^ in a cycle of austerity. Calls^ix^ for a special coronavirus wealth tax to raise funds from those who will profit from the crisis, and those by African leaders for debts to be cancelled, are initiatives to deal with COVID-19 in a way that breaks or at the very least does not exacerbate this harmful cycle. True pursuit of the right to health and fulfilment of oversight responsibilities over the private sector will require commitment to eliminating structural inequality created by prevalent global economic arrangements – this includes undertaking significant reforms. The implementation of a multi-level and multi-sectoral approach is key to aligning increasingly privatised healthcare with human rights standards, upholding the principle of progressive realisation of the right to health and holding global economic institutions to account for their approaches to funding healthcare provision.

As long as this approach does not take hold, spectators from all sides will witness the growing expenditure on healthcare not resulting in equitable improvements in access. The consequence will be that Sub-Saharan Africa will continue to have worse outcomes when dealing with pandemics, causing loss of life and inhibiting development. As stated^14^ by the UN Secretary General; ‘when we get past this crisis, which we will, we will face a choice; we can go back to the world as it was before or deal decisively with those issues that make us all unnecessarily vulnerable to crises’. All of us, particularly the medical sector, are gifted with a unique opportunity to re-imagine and work towards a world where key investment decisions are made on the basis of human and environmental well-being, not profit. Amongst others, the health sector reforms we can advocate for include:

- Generating contextually relevant evidence for health financing policy development and implementation prior to increasing the percentage of private healthcare financing, focussing on economic efficiency without negatively impacting equity.^5^
- Incentivising private health insurers to distribute the cost of healthcare across groups with heterogeneous health profiles.
- Reducing out-of-pocket payments with the view of eliminating them.
- Requiring private healthcare financing to provide desegregated data on healthcare outcomes to mitigate the effects of fee-for-service billing models and increase available information on the necessity and effectiveness of treatments.
- Planning structured and standardised schemes to allow for equitable comparison on the quality of healthcare services to improve decision-making for healthcare users, and to increase competition in the funder’s market.
- Learning from COVID-19 to create holistic and joint private–public approaches to develop a unified strong regulatory capacity and infrastructure over the entire healthcare sector which monitors competitiveness and ensures adherence to human rights responsibilities.

Let us take heart from those engaged in this work already, realise our agency to add our efforts to theirs and build a society that is truly healthy.
